# Room-Temperature Self-Standing Cellulose-Based Hydrogel Electrolytes for Electrochemical Devices

**DOI:** 10.3390/polym12112686

**Published:** 2020-11-13

**Authors:** Iñaki Gomez, Yolanda Alesanco, Jose Alberto Blázquez, Ana Viñuales, Luis C. Colmenares

**Affiliations:** CIDETEC, Basque Research and Technology Alliance (BRTA), Paseo Miramón 196, 20014 Donostia-San Sebastián, Spain; yalesanco@cidetec.es (Y.A.); ablazquez@cidetec.es (J.A.B.); avinuales@cidetec.es (A.V.)

**Keywords:** cellulose, hydrogel, electrolyte, room-temperature, electrochemical devices, electrochromic devices, zinc aqueous batteries

## Abstract

The trend of research towards more sustainable materials is pushing the application of biopolymers in a variety of unexplored fields. In this regard, hydrogels are attracting significant attention as electrolytes for flexible electrochemical devices thanks to their combination of ionic conductivity and mechanical properties. In this context, we present the use of cellulose-based hydrogels as aqueous electrolytes for electrochemical devices. These materials were obtained by crosslinking of hydroxyethyl cellulose (HEC) with divinyl sulfone (DVS) in the presence of carboxymethyl cellulose (CMC), creating a semi-IPN structure. The reaction was confirmed by NMR and FTIR. The small-amplitude oscillatory shear (SAOS) technique revealed that the rheological properties could be conveniently varied by simply changing the gel composition. Additionally, the hydrogels presented high ionic conductivity in the range of mS cm^−1^. The ease of synthesis and processing of the hydrogels allowed the assembly of an all-in-one electrochromic device (ECD) with high transmittance variation, improved switching time and good color efficiency. On the other hand, the swelling ability of the hydrogels permits the tuning of the electrolyte to improve the performance of a printed Zinc/MnO_2_ primary battery. The results prove the potential of cellulose-based hydrogels as electrolytes for more sustainable electrochemical devices.

## 1. Introduction

Wearable electronics with specific features (miniaturizing, flexibility and custom shape) are of increasing interest in applications such as medical sensing devices and smart labels in the frame of the rising of IoT [1]. These systems include several flexible electrochemical devices (i.e., sensors, batteries, electrochromic, electronics), whose performance is not affected under different mechanical stresses [2]. These technologies rely on soft and flexible materials where hydrogels have shown up as promising electrolyte systems [3,4]. These materials consist of tridimensional polymer networks that can accommodate a large amount of water within the interstices of the structure, mainly through polar or ionic bonding from functional groups in the backbone [5,6]. In this way, hydrogels combine the features of aqueous electrolytes (ionic conductivity and interfacial contact) with the mechanical properties of polymers, while preventing the principal drawbacks of aqueous electrolytes, namely the risk of leakage and fast evaporation. Besides, the versatility of polymer chemistry can confer advanced functionalities to hydrogels (i.e., improved water retention, higher ionic conductivity, rubbery stretchability, self-healing ability) to obtain engineered materials for specific applications [5,6]. All in all, the most widely used polymeric materials as a hydrogel matrix are composed of synthetic monomers from non-renewable sources. This issue, together with the increasingly worrying E-waste problem, has boosted the research on more sustainable materials for electrochemical devices [7]. In this regard, bio-source polymers offer a variety of materials with a wide range of different characteristics, such as hydrophilicity, good mechanical properties and easy chemical modification ability. These features, summed to their inherent biodegradability, and their high abundance makes biopolymers a suitable choice for sustainable hydrogel electrolytes [8,9,10].

Among the large variety of biomaterials that nature offers, cellulose is the most abundant biopolymer on Earth, present as one of the main structural components of plants and seaweeds. Composed of D-anhydroglucopyranose repeating units linked by 1,4-β glycosidic bonds, it possesses a high amount of hydroxyl groups in the linear polymeric backbone that confers several characteristics, such as high mechanical properties, chemical modification ability and high hydrophilicity [11]. These features, summed with the inherent biocompatibility and biodegradability of cellulose, have promoted tremendous research on cellulose-based hydrogels (CBH) in health sciences such as tissue engineering, drug delivery and wound dressing [12]. Besides, CBHs based on CMC present superabsorbent behavior, promoting their use in applications such as water cleaning, water reservoirs for agriculture and personal hygiene items [8,13]. Despite the benefits of cellulose as a hydrogel matrix, the use of CBHs in electrochemical devices as electrolytes is relatively scarce. However, the trend of research in materials and devices towards a circular economy is pushing the application of biopolymers in a variety of unexplored field [14].

The high amount of intra- and intermolecular H-bonds between macromolecules promotes strong crystalline structures, making native cellulose an insoluble and intractable material [15]. Specific solvent systems can effectively break these interactions and dissolve cellulose, which allows for its processing. For instance, Kasprzak and coworkers used an ionic liquid/DMSO solvent system to dissolve cellulose to fabricate both hydrogel electrolytes and composite electrodes for an electrochemical double-layer capacitors (EDLC) [16]. More recently, Yan and coworkers presented flexible capacitors by coagulating a cellulose solution in ZnCl_2_ water-in-salt media [17]. Through these strategies, the main structural support relies on hydrophobic interaction and/or entanglement forces that confer limited mechanical stability. In this regard, Navarra and coworkers studied the feasibility of CBHs as electrolytes obtained through the crosslinking of a cellulose solution in a NaOH/urea system with epichlorhydrin [18]. More recently, Yan and coworkers used this system as an electrolyte for an asymmetric capacitor, showing that high energy density as well as high power capacity were achieved [19]. The main drawback of these approaches relies on the liquid media needed for the dissolution of cellulose, which is unsuitable for alternative electrochemical devices such as batteries and/or electrochromic (ECDs). In this regard, cellulose derivatives offer a variety of properties thanks to their functional groups introduced through the modification of the hydroxyl groups of the backbone. In this way, several water-soluble cellulosic materials can be obtained such as methyl cellulose (MC), hydroxyethyl cellulose (HEC) and carboxymethyl cellulose (CMC). Shi and coworkers took advantage of the hydrophobic interactions of methyl cellulose (MC) and hydroxypropyl cellulose (HPC) in order to obtain thermoresponsive electrolytes for safer energy storage devices [20]. Gajewski and Béguin proved that a CBH based on CMC improved the performance of electrochemical capacitors when comparing with commercial separators such as Celgard or Whatman [21]. However, these materials lack dimensional stability, which hinders their potential use for flexible devices.

In this context, we present the synthesis of self-standing cellulose-based hydrogels and their application as electrolytes for electrochemical devices. These materials were obtained through the crosslinking reaction of cellulose derivatives with divinyl sulfone (DVS) in aqueous media. This approach has been extensively reported as an effective method to obtain hydrogels for tissue engineering, biomedicine and bioelectronics [22,23,24,25]. All in all, this work gives a deeper insight into the impact of the material composition on the rheological parameters. Moreover, this is the first report to explore the potential of these hydrogels as electrolytes for electrochemical devices. In this regard, the ease of hydrogel synthesis favors the assembly of all-in-one electrochromic devices, and their swelling behavior allows adjusting the electrolytic solution for a printed aqueous zinc battery. These results show the potential of CBH as electrolytes for a variety of electrochemical devices as well as processing methods such as tape casting or printing techniques.

## 2. Materials and Methods

### 2.1. Materials

Carboxy methyl cellulose (DuPont, Wilmington, DE, USA. 2000 Pa, DS: 0.82–0.95), hydroxyethyl cellulose (Natrosol^TM^, Ashland Global, Covington, KY, USA), divinyl sulfone (TCI chemicals, Tokyo, Japan, 96%), ethyl viologen dibromide (Sigma-Aldrich, St. Louis, MO, USA 99%), potassium ferrocyanide (Sigma-Aldrich, 98.5%), potassium ferricyanide (Sigma-Aldrich, 99%) and ZnCl_2_ (Sigma-Aldrich, >98%) were used without further purification.

### 2.2. Synthesis of Cellulose-Based Hydrogel (General Procedure)

The precursor of the cellulose-based hydrogel was prepared by dissolving the proper amount of CMC and HEC in 9.4 g of a KOH solution. This cellulose-based precursor solution was stirred until a viscous, clear and transparent medium was obtained. Subsequently, DVS was added and the solution was stirred until gelification of the media was observed.

### 2.3. Characterization Methods

The NMR spectra were recorded in a Bruker 500 MHz spectrometer with deuterated water as solvent. For the hydrogel, 0.01 g of HEC was dissolved in 10 mM KOH followed by the addition of 0.01 g of DVS. The mixture was quickly placed into the NMR tube for the full reaction.

The ATR-FTIR spectra were obtained in the wavenumber range from 4000 to 400 cm^−1^ over 64 scans with a resolution of 4 cm^−1^ using a 4100LE FTIR (Jasco, Hachioji-shi, Tokyo, Japan). The samples were dried in the oven until constant weight.

Rheological measurements were carried out in a TA instruments AR2000ex Rheometer employing parallel plates of 40 mm diameter. Time sweep experiments were performed with frequency and strain values set at 1 Hz and 1%, respectively. Temperature was kept at 25 °C. For frequency and strain sweeps, the hydrogels were cured in the parallel plates at 25 °C. Strain sweeps were performed at a frequency of 1 Hz in a range of strains from 0.01% to 100%. Frequency sweeps were performed at a strain of 1% in a range of 0.001 to 100 Hz.

Ionic conductivity of hydrogels was determined by electrochemical impedance spectroscopy (EIS) in a potentiostat Solartron Analytics (Farnborough, UK) with a CR2032 coin cell setup. The precursor solution of the desired hydrogel electrolyte was placed between two stainless steel disks, separated by a PET ring (thickness of 0.4 mm; inner diameter of 11 mm). After the coin cell was sealed, the hydrogel electrolytes were allowed to react for 90 min. Electrochromic properties of the ECDs were evaluated with a Jasco V-570 UV-Vis Spectrophotometer (Jasco, Hachioji-shi, Tokyo, Japan) provided with a films holder accessory for solid samples, while the devices were connected to a Biologic MPG potentiostat/galvanostat (BioLogic Science Instruments, Auvergne-Rhône-Alpe, France) as a direct current source. UV–Vis spectra were obtained in transmission mode using air as the background.

The photographs of the ECDs were acquired using a Canon IXUS 105 camera while the ECDs were lit with a fluorescent light source (Phillips TL-D 80 18W/840), without the employment of flash (focal distance = 5 mm, maximum opening = 3 and exposition time = 30–50 s).

The printed batteries were tested in a Biologic MPG potentiostat/galvanostat. The galvanostatic discharge experiments were performed at 50 and 100 µA. For the intermittent discharge experiment, power pulses of 100 µW for 6 s and 30 s of resting in open circuit potential (OCV) were applied.

### 2.4. Assembly of All-in-One ECDs

To a cellulose-based precursor solution (CMC, HEC and KOH solution), ethyl viologen dibromide (20 mM) and a 1:1 mixture of potassium ferrocyanide and ferricyanide (6 mM) were added. This formulation was stirred until a homogeneous solution was obtained. DVS was purified by passing it through the inhibitor remover column. Detailed information regarding the assembly process of all-in-one ECDs can be found in previously published work [26]. Briefly, the cellulose-based electrochromic hydrogel was spread on the ITO-coated side of one of the ITO-PET substrates provided with a spacer along the whole perimeter (double-sided adhesive tape frame of 220 µm thick and 3 mm wide). Then, it was covered with the other ITO-PET electrode substrate, applying light pressure in order to guarantee the adhesion between both electrodes.

### 2.5. Assembly of Printed Batteries

Printed zinc/MnO_2_ batteries were kindly supplied by Varta Microbattery GmbH (Ellwangen, Germany). A precursor of cellulose-based electrolytes (0.2 g of CMC, 0.2 g of HEC and 0.2 g of DVS in 9.4 mL of 20 mM of KOH) was casted onto the electrodes using a custom-made double-taped mask. The solution was allowed to react for 90 min. Afterwards, the battery was sealed with another homemade mask. For printed batteries tested with the ZnCl_2_ electrolyte, the battery’s electrodes were coated with the cellulose-based electrolyte. After 90 min of reaction, the coated battery was dried in an oven (30 °C) until constant weight. Finally, the film was swelled, adding a given volume of 1 M of ZnCl_2_ electrolyte, and then the battery was sealed.

## 3. Results and Discussion

### 3.1. Synthesis and Physicochemical Characterization

**Figure 1:**
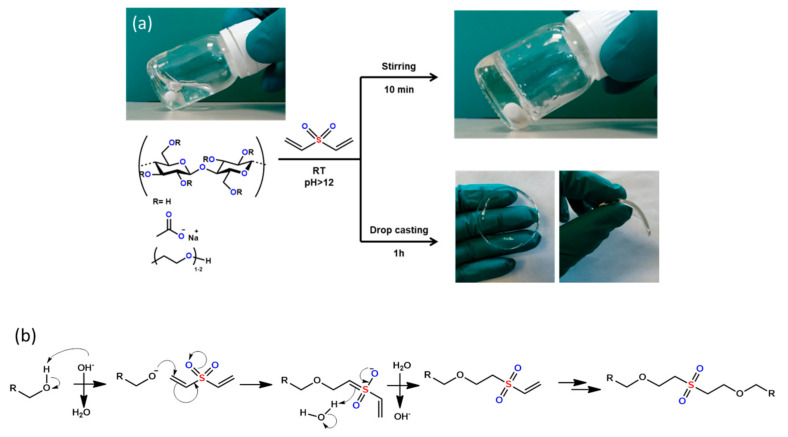
(**a**) Schematic representation of the synthesis of cellulose-based hydrogels (**b**) and crosslinking reaction mechanism.

Cellulose-based hydrogels (CBH) were obtained by the crosslinking of cellulose derivatives with divinyl sulfone (DVS) at room temperature. The reaction proceeded by the dissolution of carboxymethyl cellulose (CMC) and hydroxyethyl cellulose (HEC) in alkaline aqueous media (12 < pH < 13), followed by the addition of the crosslinking agent (DVS). After a short period of time (ca. 10 min) stirring, the reaction media solidified, obtaining a transparent gel. Self-standing transparent and soft hydrogel films were obtained by drop casting the precursor solution upon a Petri dish and leaving it for 60 min (Figure 1a). The crosslinking mechanism involved the deprotonation of the hydroxyl groups of the cellulose derivatives induced by the pH of the media and the subsequent attack of the alkoxy anions on the electron-deficient vinyl groups from the DVS [27] (Figure 1b).

**Figure 2:**
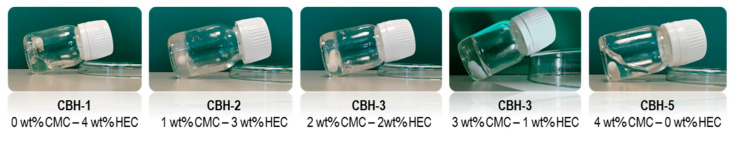
Digital images of gel appearance as a function of the cellulose derivatives’ weight ratio. CBH is the abbreviation for cellulose-based hydrogel; CMC for carboxymethyl cellulose and HEC for hydroxyethyl cellulose.

The effect of the cellulose derivatives’ weight ratio (hydrogel composition) in the crosslinking reaction was investigated (Table 1). When HEC was used as the only cellulose source (CBH-1), the obtained material was a tough, solid gel that released water from its structure. In order to improve the water retention of CMC, an anionic cellulose derivative was added to the formulation. The gels CBH-2, CBH-3 and CBH-4, with an increased concentration of CMC, were found to be softer gels that did not release a significant amount of water (Figure 2). In an attempt to have the maximum amount of ionic moieties in the gel matrix, the crosslinking reaction was tried with only CMC as the cellulose source, but no gel was formed (CBH-5 in Figure 2).

It has already been reported that CMC shows no reactivity towards DVS since the reactants of the OH^−^ groups in position C6 are, to a large extent, replaced by carboxyl groups, and the other hydroxyl groups present in the cellulose backbone (C2 and C3) are sterically hindered [28,29]. This fact points out that, when both cellulose derivatives are present, only HEC takes part in the crosslinking reaction with DVS (Figure 1b). This process leads to a structure so-called the semi-interpenetrating polymer network (semi-IPN), where a linear polymer is imbibed in a tridimensional crosslinked network [6]. This structure enables combining the functionality of a linear polymer with the mechanical properties provided by the crosslinked material—in this case, the water retention ability of the CMC polymer with the gel forming ability of the HEC–DVS system.

**Figure 3:**
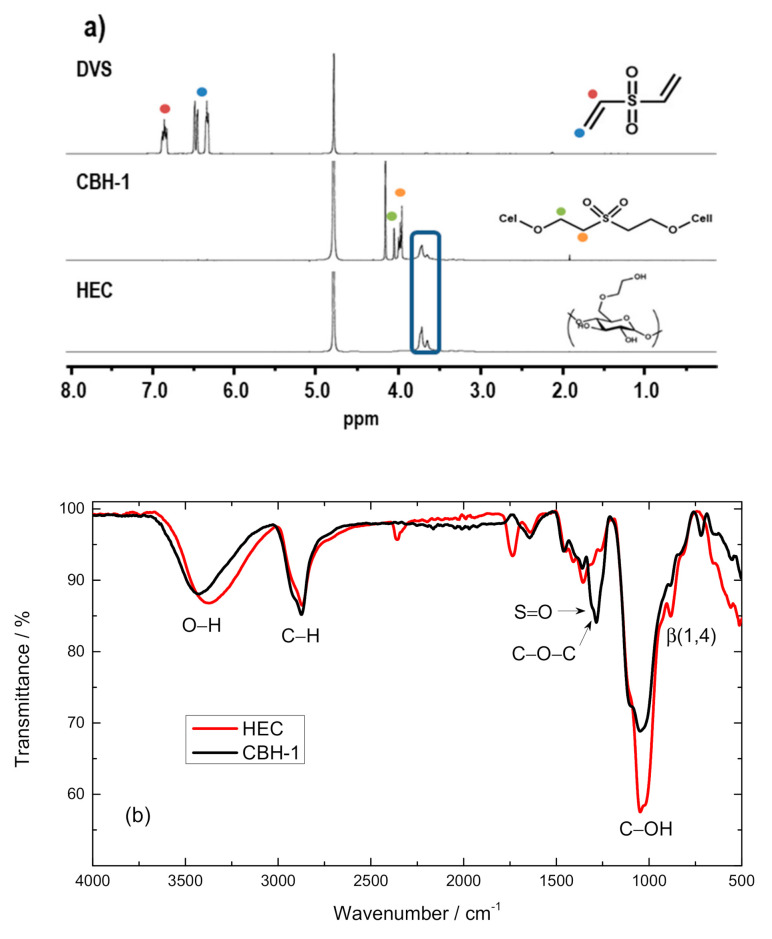
Characterization of the crosslinking reaction: (**a**) H^1^ NMR spectra of divinyl sulfone (DVS) (top); CBH-1 (middle) and HEC (bottom); (**b**) spectra of HEC (red) and CBH-1 (black).

The chemical characterization was performed over the hydrogel obtained with HEC as the only cellulose source in order to avoid signals from CMC that could interfere with the signals of the reaction. Figure 3a displays the ^1^H NMR spectra of DVS, HEC and the formed hydrogel (CBH-1). DVS spectra are characterized by the signal of the double bounds at 6.4 and 7.0 ppm (blue and red circles, respectively). These signals are not present in CBH-1 spectra. This result confirms the total consumption of DVS during the HEC crosslinking. Furthermore, CBH-1 spectra show new bands at 3.9 and 4.1 ppm (orange and green circles, respectively), which are assigned to the protons bonded to the sulfonyl group. In contrast, the signals (3.6–3.8 ppm) from the HEC backbone remain unaltered (region marked by the blue line in Figure 3a), as demonstrated by the HEC spectra.

To further confirm the crosslinking reaction, ATR-FTIR spectra of HEC and CBH-1 (dried film of the hydrogel HEC–DVS) were measured (Figure 3b). The bands related to the HEC backbone, namely the O-H stretching vibration (3500 cm^−1^), C-H stretching vibration (2950 cm^−1^), C-H and O-H bending vibrations (1400–1100 cm^−1^) and the band related to the β(1-4) glycosidic bond (920 cm^−1^), remain unaltered (red line) in the CBH-1 spectrum (black line) [30,31]. For CBH-1, a new broad band with a shoulder at 1310 cm^−1^ and the peak of the band at 1280 cm^−1^ appears. This band is assigned to the S=O stretching vibration of the sulfone moiety and the ether bond (C-O-C) between HEC and DVS [32]. This confirms the successful reaction between DVS and HEC.

### 3.2. Rheological Characterization

The gel properties were studied by the small-amplitude oscillatory shear (SAOS) rheological technique. For these measurements, the preparation of the sample is essential [33]. If the hydrogel is prepared ex situ, when placed in the rheometer geometry, the structure might be stressed by the pressure of the plate, disturbing the results. Therefore, the samples were placed in their liquid state and gelled in situ to the parallel plates at room temperature. The protocol set by Morrison and coworkers establishes that firstly an approximated gel time has to be determined by performing a time sweep, where arbitrary values of strain and frequency are set [33]. Taking previous reports on CBH formed by the HEC–DVS system, a strain of 1% and frequency of 1 Hz were chosen [27]. Afterwards, the linear viscoelastic regime (LVE) and linear modulus plateau (LMP) were studied by strain and frequency sweeps. In this way, a systematic study of the effect of KOH concentration and HEC to DVS wt. % ratio (Table 2) on the rheological properties of the hydrogels was performed.

The rheological characterization of hydrogel CBH-3 is displayed in Figure S1 as a representative study for every single gel investigated. The outcomes are summarized in Table 2 for all CBHs. Figure S1a illustrates the time sweep experiment, where initially the loss modulus (G″; dashed line) is above the storage modulus (G′; solid line). This means that the sample has a viscous (liquid) behavior. The point when the G′ line crosses the G″ line is assigned as the gelling time. For CBH-3, it was 12 min. It is worth mentioning that the storage modulus continues to increase over a period of 80 min. This suggests that the sol–gel transition happens at the beginning of the crosslinking reaction. Consequently, taking this information into account, the strain and frequency sweeps were performed after allowing the gel to react for 90 min.

The viscoelastic behavior was studied by applying a strain sweep at a frequency of 1 Hz (Figure S1b). The LVE region covers from 0.01% to 20% of the strain, where the storage modulus (G′) suffers a deviation from the linearity. This indicates that the sample starts to flow, suggesting that the tridimensional network responsible of the elastic behavior started to break.

Additionally, the frequency sweep was performed at a strain of 1% in a range of 0.001 to 100 Hz (Figure S1c). CBH-3 presents an LMP up to 50 Hz. The results point out that the time sweep performed at 1% of strain and 1 Hz is within the LVE and LMP ranges. These results reinforce that the value obtained for the sol–gel transition can be taken as valid. Therefore, the sol–gel transition values were then established for characterizing all the gels (gel time in Table 2).

**Figure 4:**
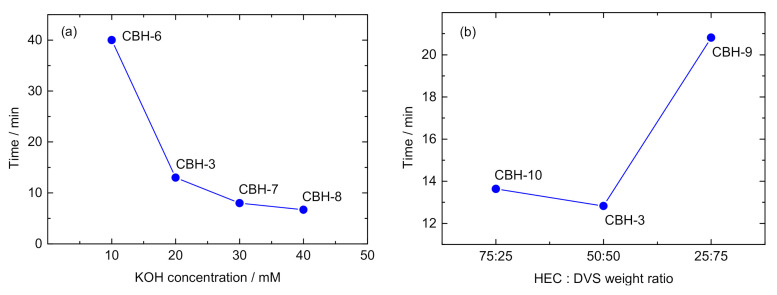
Sol–gel transition of hydrogels depending on (**a**) concentration of KOH and (**b**) HEC–DVS weight ratio.

The dependence of the gel time (sol–gel transition) on the KOH concentration and HEC–DVS wt. % ratio is displayed in Figure 4a,b, respectively. As noticed, increasing the KOH concentration provokes a faster sol–gel transition, as shown in Figure 4a and reported in Table 2. It is understood as an increment in the crosslinking kinetics induced by the higher concentration of alkoxy anions when the KOH concentration rises. This may lead to a higher reactivity of HEC. In contrast, when the ratio HEC–DVS increases at the same KOH concentration, it results in a lower gel time (viz., 21 min for CBH-9, 12 min for CHB-3 and 13 min for CBH-10). This faster crosslinking kinetics is then associated with the higher HEC content as the reactive compound.

Additionally, at a low-frequency range, the constant modulus (G′) can be correlated with the mesh size. Thus, through the simple rubber elasticity theory (Equation (1)), it was calculated for each hydrogel (Table 2) [34]:

(1) $$\xi =\sqrt[{3}]{\frac{k_{B}T}{G^{\prime }}}$$

where *ξ* is the mesh size, *k_B_* is the Boltzmann constant and *T* is the temperature.

After equilibration of the crosslinking reaction, the hydrogels were subjected to strain and frequency sweeps (Figures S2 and S3). As summarized in Table 2, the modulus (G′) of the hydrogels and the mesh sizes (ξ) show a strong dependency on the reaction conditions. As reported, when the KOH concentration is, e.g., four times higher (from 10 to 40 mM), G’ increases almost one order of magnitude (from 1100 for CBH-6 to 9100 Pa for CBH-8). Furthermore, the increment of KOH concentration promotes a higher crosslinking density as represented by the lower mesh size values. As previously stated, the higher concentration of hydroxide (OH^−^) in the aqueous media promotes a higher alkoxy anion amount in HEC, leading to higher crosslinking points in the macromolecular structure. Similar behavior can be observed when increasing the HEC–DVS ratio, i.e., higher modulus and lower mesh size, indicating a higher crosslinking density. In contrast, as shown, when the content of HEC in the gel is lower (i.e., 1 wt. % in CBH-9), the concentration of the reactant decreases. It leads to a low crosslinking density and therefore a lower modulus (G′ = 1100 Pa) and higher mesh size (ξ = 15.52 nm).

The viscoelastic properties of the CBHs are given by the behavior of the lineal viscoelastic regime (LVE) and the linear modulus plateau (LMP). All the hydrogels show a very similar LMP of around 50 Hz (Figures S2b and S3b). However, the LVE has a strong dependence on the KOH concentration and hence on the crosslinking density. When the hydrogel is highly crosslinked (viz., CBH-7 and CBH-8; Table 2), the viscoelastic response is lower (LVE of about 13% strain) due to a more rigid structure (Figure S2a). In contrast, at lower KOH concentration (lower crosslinking density), the ability of the tridimensional network to absorb stress (strain) increases. The latter is translated into higher LVE values for CBH-3 (38% strain) and CBH-6 (80% strain). Interestingly, the LVE regime is very similar for CBH-9 and CBH-10, suggesting that the variation in the HEC to DVS ratio has little impact in the viscoelastic properties (Figure S3a).

This comprehensive characterization of the rheological properties of cellulose-based hydrogels reveals that by tuning the formulation, parameters such as the modulus, sol–gel transition and viscoelastic properties can be adjusted. This feature may allow choosing the desired parameters of the selected processing techniques, such as printing (screen, ink-jet, 3D) or coating (dip, spin or knife) methods for a given application.

### 3.3. Ionic Conductivity

Once the crosslinking reaction and the mechanical properties were characterized, the other main feature of the gel electrolytes, namely ionic conductivity, was characterized. Similarly to the rheological measurements, the hydrogels were synthesized in situ in a coin cell for their characterization. The values of ionic conductivities are listed in Table 2. The gels possess higher ionic conductivities than their corresponding aqueous media, mainly due to the presence of a higher amount of mobile ions, namely the Na^+^ cations from the CMC. This fact points out that the ionic nature of CMC has two effects on hydrogels: on one hand, it enhances the water retention, and on the other hand, it increases the ionic conductivity of the hydrogels.

In the gels CBH-6, CBH-7 and CBH-8, the KOH aqueous solutions show an increasing trend of ionic conductivity with the concentration. However, this has little impact on the conductivity of the gels, where the values remain very similar (between 5 and 6 mS cm^−1^). In the case of CBH-9 and 10, a decrease in the ionic conductivity is observed, although the concentration of CMC and KOH remains constant. This behavior can be correlated with the results obtained in the rheological measurements (Section 3.2). In the case of the increasing the KOH concentration, although the ionic conductivity of the solution is higher, the decrease in the mesh size due to the higher crosslinking density hinders the mobility of the ions through the structure. When increasing the HEC–DVS ratio, the mesh size decreases with a consequent drop in the ionic conductivity.

Finally, the viability of this cellulose-based hydrogel as electrolytes in electrochemical devices was assessed. As a proof of concept, electrochromic displays (ECDs) and primary printed Zn/MnO_2_ batteries were chosen.

### 3.4. Electrochromic Devices (ECDs)

Among a variety of electrochromic (EC) device configurations, they can be broadly divided into two major groups: (1) layered- and (2) all-in-one-type configurations. The layered type refers to the ECD architecture wherein the chromophore is deposited on or attached to the working electrode and the electrolyte is incorporated as a discrete layer [35,36,37,38]. The all-in-one type describes a less complex device architecture in which all electroactive materials (i.e., EC materials and the redox mediators) are dissolved in the electrolyte, and the resulting EC mixture (single layer) is sandwiched between two electrode substrates in a symmetric device configuration (e.g., glass/TCO/EC mixture/TCO/glass). Apart from the predictable advantages of the all-in-one configuration (simpler assembly process, easier to industrialize), it may also exhibit other strengths, such as exceptional versatility, enhanced durability and suitability for flexible substrates [39]. In addition, the employment of these all-in-one device architectures together with gel electrolytes has been proven to stabilize some chemical substances involved in EC systems, enhancing the chromatic diversity of ECDs. In this context, the development of cellulose-based hydrogel electrolytes suitable for all-in-one ECDs may be the more environmental approach to maintain all these exceptional features [40,41]. However, the main reported devices employed layered-type configurations using rigid-glass substrates [42,43,44,45,46,47,48]. In this context, it is worth to point out that the ECDs comprising cellulose hydrogel electrolytes reported herein are based on all-in-one configurations employing flexible substrates (ITO/PET).

**Figure 5:**
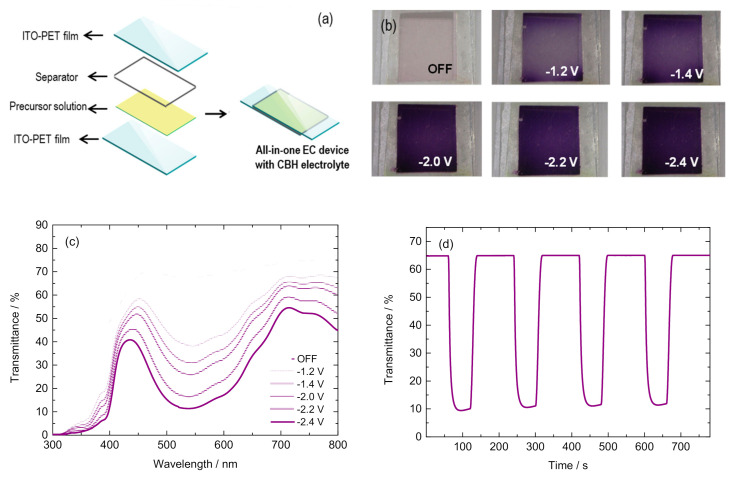
Electrochromic performance of cellulose-based hydrogels (CBHs): (**a**) schematic representation of the all-in-one electrochromic (EC) device with the flexible poly(ethylene terephtalate) coated with indium tin oxide (ITO-PET); (**b**) pictures at bleached state (OFF) and upon applying different cathodic potentials (from −2.4 to −1.2 V); (**c**) transmittance spectra at bleached state (OFF) and upon applying different cathodic potentials (from −2.4 to −1.2 V); (**d**) transmittance changes (at 550 nm) vs. time with square wave potential steps between bleached (0 V for 120 s) and colored state (−2.4 V for 60 s).

An optimized cellulose-based EC hydrogel: 20 mM of EtVio as the EC material and 6 mM of Ferro/Ferri as the redox mediator, was assembled in all-in-one architecture with the CBH-3 composition (Figure 5a). It is worth mentioning that the commercial DVS is stabilized with hydroquinone (HQ). In order to avoid the possible effect of HQ on the electrochromic behavior, DVS was purified by passing it through the inhibitor remover column. Aiming to promote a concentration gradient of the viologen molecules on the cathode/electrolyte interface, devices were exposed to a suitable cathodic potential for a longer time than that required for the sol–gel transition to occur (i.e., −1.0 V for 15 min.). Afterward, the EC performance of these ECDs was conveniently evaluated to assess the suitability of the cellulose hydrogel reported herein as a potential electrolyte.

Two main parameters to evaluate the electrochromic behavior of EC devices are the level of coloration and the transmittance changes between the colored and bleached states. The devices showed a transparent colorless off-state and acquired a weak bluish coloration upon applying −1.2 V (Figure 5b). As the cathodic potential increased, the transmittance level of the device diminished, providing ECDs with higher levels of coloration. The level of coloration of the devices was evaluated through UV–visible transmittance spectra for different applied potentials (Figure 5c). The percentage of transmittance (%T) at the maximum contrast wavelength (550 nm) reached a value of 12% when the device was exposed to −2.4 V (Table S1). The transmittance changes (Δ%T), defined as the difference between the transmittance in the bleached state and that obtained in the colored state, ranged from 29.4% to 55.8% for −1.2 and −2.4 V applied potentials, respectively, thus providing ECDs with modulated levels of coloration.

With regard to the colorations displayed by the ECDs at both colorless off-state and colored state (−2.4 V), an unequivocal interpretation of them was numerically determined through the transmittance profiles of the ECDs and using a spectrophotometric method previously reported (Table S2) [49,50]. These data confirmed the absence of color (a* and b* chromaticity coordinates very close to 0 value) and the excellent transparency (*L 86) of the ECDs at off-state. Upon applying −2.4 V cathodic potential, color coordinates revealed a significant decrease in the lightness (*L 46), in agreement with the high Δ%T obtained for this voltage. Noteworthy variation was also observed for chromaticity coordinates at the stated cathodic potential. Thus, a* and b* color coordinates acquired 32 and −31 values, respectively, revealing red and blue characters of the resultant purple-colored state. This coloration observed in ECDs based on alkyl viologens, such as EtVio employed herein, is attributed to the well-studied phenomenon of the radical cation monomer (blue) and its dimer formation (red), more likely to occur when using aqueous electrolytic systems [26,51].

Another key parameter for assessing the electrochromic behavior of EC devices is the switching time, denoting the time required to observe the transition between the colored and bleached states. The devices were exposed long enough to square-wave potential steps between bleached (0 V) and colored states (−2.4 V) while the corresponding transmittance changes were being recorded in the spectrophotometer over time at a fixed wavelength of 550 nm (Figure 5d). These studies revealed switching times of 11 s for both coloring (tc) and bleaching processes (tb), estimated as the time required to reach 90% of the total Δ%T for the colored and bleached steps.

With regard to the power efficiency of the devices, it is usually quantified through their color efficiencies (η). The latter parameter, correlated with the electrode area, is defined as the change in optical density during the redox process at a given wavelength divided by the injected/ejected charge [45]. The ECDs based on the cellulose hydrogel reported herein exhibited color efficiencies of 122 and 218 cm^2^ C^−1^ for coloring (ηc) and bleaching processes (ηb), respectively (λ = 550 nm).

In order to assess the flexibility of the devices and therefore their feasibility to be adapted to non-flat surfaces, the ECDs were exposed to bending tests. The coloration of the devices remained homogeneous during the bending process due to the self-standing character of the electrolyte that guarantees a regular distribution of it throughout the device area (Figure S4a). Further bending studies confirmed the stability of the ECDs, with no decay of their coloration or ∆%T and no visual signs of deterioration after 50 cycles of bending (Figure S4b,c and Table S3).

When comparing these results with the little available reports on other all-in-one EC systems based on viologens and comprising cellulose derivative electrolytes, some conclusions can be drawn. The maximum transmittance variation achieved for the flexible EC system reported in the present work (i.e., 55.8%) is in the range of that obtained for rigid-glass devices and overruns that reported for flexible plastic substrates. The switching times of the EC system described herein were in the range of the latter, while the color efficiencies were close to the former [47,52]. These results prove that the room-temperature self-standing cellulose hydrogel employed in the present work may be a potential electrolyte for all-in-one ECDs.

### 3.5. Batteries

Nowadays, among others, devices like wireless sensors, smart labels, medical strips, printed electronics for different applications such as the Internet of Things (IoT) and wearable electronics need power in the range of microwatts (µW) to milliwatts (mW), and capacities from microamp-hours (µAh) to hundreds of milliamp-hours (mAh), depending on the final application. This need has driven the development of thin, small, low-cost and environmentally friendly batteries as a power source. Battery manufactures look for simplifying the process to achieve lower cost, quality and mass production. Furthermore, if manufacturing includes a low-temperature process, it will enable the use of flexible substrates and more environmentally friendly materials like, e.g., paper. In that regard, printing technologies seem to be an attractive alternative for manufacturing such type of batteries, promising a cheap mass production while the need for the customization or design of the batteries to meet the specific requirements of the final application is assured. Besides, printing technologies enable advanced large-scale manufacturing processes of batteries, such as printing into integrated platforms and/or onto soft substrates for flexible and stretchable devices [53,54]

Aqueous batteries are an attractive option as sustainable and safe technologies for energy storage in several applications, as mentioned above. Consequently, zinc-based battery technologies show up as promising candidates thanks to the electrochemical properties and environmental friendliness of zinc [55]. On the other hand, the use of hydrogel electrolytes has boosted the research of these systems in flexible and/or printed applications [56]. However, the main used materials for those implemented in hydrogel electrolyte synthesis rely on synthetic polymers such as poly(vinyl alcohol) (PVA), poly(acrylic acid) (PAA) and/or poly(acrylamide) (PAM) [57]. In order to move towards a more sustainable system, the use of cellulose-based hydrogels as electrolytes is an attractive alternative.

Taking into account the aspects mentioned above, this section focuses on a primary (not rechargeable) printed battery using traditional zinc/manganese dioxide chemistry manufactured and kindly provided by Varta Microbattery GmbH. With this battery, the here proposed hydrogel electrolyte approach will be evaluated as a function of the battery performance. Figure S5 illustrates a schematic representation of the battery printed on a paper substrate. The battery has a so-called co-planar (also known as side-by-side) configuration. The black square represents the positive electrode composed of carbon and MnO_2_ and the gray square exemplifies the negative electrode based on zinc. The commonly used electrolytes are a solution of zinc chloride (ZnCl_2_) or KOH from which the battery is known as a zinc-chloride or alkaline battery, respectively. The conventional and commercial available battery format of this chemistry is usually recommended for low-drain devices since they are a very reliable source of power on both continuous and intermittent discharges and when cost is an important consideration. This battery chemistry has usually a nominal voltage of about 1.5 V.

**Figure 6:**
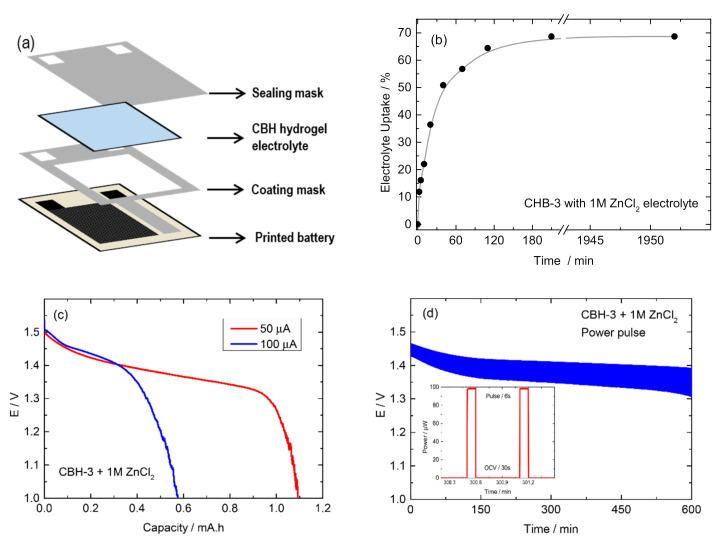
(**a**) Schematic representation of the coating procedure of cellulose-based hydrogel upon printed battery; (**b**) swelling behavior of CBH with 1 M ZnCl_2_ electrolyte; (**c**) galvanostatic discharge curves of the printed battery with CBH-3 swelled in 1 M ZnCl_2;_ (**d**) intermittent discharge profile via power pulses (6 s: 100 µW; 30 s: open circuit potential (OCV)) of the battery with CBH-3 swelled in 1 M ZnCl_2_.

Figure 6a represents the sequence implemented for casting (coating mask) the hydrogel electrolyte and subsequently sealing (sealing mask) the battery. The hydrogel CBH-3 (20 mM KOH) was casted upon the electrodes, left to react for about 90 min and sealed (see Section 3.2) before electrochemically assessing the battery. The open circuit potential (OCV) for this battery was around 1.26 V, which is slightly lower than the usually reported value for alkaline batteries (i.e., 1.43 V for Zn(s) + 2MnO_2_(s) → ZnO(s) + Mn_2_O_3_(s)). This deviation could be due to side reactions happening on the Zn surface promoting the formation of zincates like [Zn(OH)_3_]^−^ and [Zn(OH)_4_]^2−^ which have an equilibrium potential about −1.15 V and −1.199 V, respectively. At the positive electrode, MnO_2_ is usually in equilibrium with Mn_2_O_3_ (Eº = 0.15 V). Thus, the overall cell potential might be expected to be around 1.3 and 1.35 V. Figure S6 shows the discharge profile of CBH-3 at two discharge currents (50 and 100 µA). The curves fall with a very steep slope that can be assigned to the low rate capability of the battery with CBH-3. It could be assigned to the high resistance (113 Ohm from Figure S7) and/or to the fast passivation of the zinc surface in this configuration where insoluble ZnO(s) may occur, preventing the maximal utilization of the zinc electrode. The battery provided in both cases about 90 µAh of capacity up to 1.0 V. The latter could be also assigned to the limited accessibility of the active materials (zinc and manganese dioxide) to the in situ generated hydrogel electrolyte.

In order to access the versatility of implementing hydrogel electrolytes and to evaluate the electrochemical performance of the battery, another electrolyte system based on ZnCl_2_ was introduced to the CBH. Initially the addition of Zn salt was tried by the one-pot approach, similarly to the previous experiments of ECDs. However, the presence of Zn cations was not compatible with this system (Figure S8a). On one hand, zinc reacted with the hydroxide present in the media, forming oxides. Consequently, this reaction consumes OH^−^, provoking a decrease in the pH. On the other hand, a phase separation and precipitation of the system was observed, driven by the ionic interaction of the divalent Zn^2+^ cations with the negatively charged CMC (Figure S8b). Alternatively, in order to obtain a hydrogel containing the ZnCl_2_ electrolyte, it took advantage of the superabsorbent properties of the cellulose-based hydrogels [29]. In this regard, a precursor solution of CHB-3 was casted onto a glass substrate and covered with a Petri dish in order to avoid a fast evaporation of DVS for 90 min. Then, the formed self-standing hydrogel was dried under mild conditions in an oven at 30 °C until constant weight. Afterwards, the resulting film was immersed into a solution of 1M ZnCl_2_. The increase in weight was monitored over time, showing a maximum electrolyte uptake of 70 wt. % of the dried gel weight after 180 min (Figure 6b). It is worth mentioning that the hydrogel membrane maintained the mechanical stability and shape after the swelling process (Figure S9).

After setting the swelling procedure of CBH-3 with the ZnCl_2_ solution, CBH-3 was coated upon the battery. After film drying, it was determined that the weight of the cellulose-based film needed to cover the whole battery (~14 cm^2^) was as low as 100 mg (ca. 7 mg cm^−2^). A given volume of 1 M ZnCl_2_ solution was added to the dried film, ensuring the wetting of the whole area of the battery, and then sealed (Figure 6a). In order to monitor the swelling of the hydrogel, the OCV of the battery was measured over time until a maximum of 1.52 V was achieved after 30 min. The battery cells with CBH-3 swelled in ZnCl_2_ were discharged at a constant current of 50 and 100 µA (Figure 6c). As noticed, up to 1.0 V (cut-off voltage), the battery delivered a nominal capacity as high as 1100 µAh (viz., 11.31 mAh g^−1^ Zn; 0.08 mAh cm^−2^; 5.67 Ah cm^−3^) at 50 µA. This value is more than one order of magnitude in comparison to that for the CBH-3 electrolyte. This reflects that the electrochemical conversion of the zinc surface is more efficient as long as the active surface of the metal is exposed properly to the electrolyte which is achieved with the use of the swelled CBH-3 film. Thus, the difference in the capacity can be assigned to the limited contact of the active material with the CBH-3 electrolyte. Furthermore, due to the mild acid pH (5–6) of the hydrogel electrolyte with ZnCl_2_, the zinc undergoes a conversion to Zn^2+^ without the formation of any passivation layer upon the metal surface. The overall capacity delivered by the battery coated with the hydrogel is lower (37% less) than that for the battery discharged using the liquid 1 M ZnCl_2_ electrolyte (i.e., 1740 µAh in Figure S10). Nevertheless, it is worth mentioning that the voltage profiles for both are very similar. This reveals that the battery coated with the CBH-3 hydrogel swelled in 1M ZnCl_2_ can provide the required power demanded by, e.g., smart labels or point-of-care devices.

As mentioned above, some IoT devices require power in the range of microwatts in an intermittent manner, thus the printed battery coated with CBH-3 swelled in ZnCl_2_ was evaluated under such conditions by applying an intermittent discharge protocol demanding, for 6 s, a power of 100 µW for every 30 s (Figure 6d). As shown in Figure 6d, the battery was able to deliver about 1400 times the required power before reaching the cut-off voltage of 1.0 V. These results prove that the room-temperature self-standing cellulose hydrogel could be implemented in power supply devices by adjusting its properties accordingly to the application of the device.

## 4. Conclusions

The present work points out the potential of cellulose-based hydrogels (CBH) as electrolytes for electrochemical devices. The synthesis pathway involved a well-known method to crosslink cellulose derivatives in aqueous media with divinyl sulfone (DVS). The gels were formed through a semi-IPN structure where the 3D network was formed by crosslinked hydroxyethyl cellulose (HEC) and DVS, including carboxymethyl cellulose (CMC) to enhance the water retention of the gel owing to its ionic nature. The systematic rheological characterization revealed that the gelling time, the modulus and the viscoelastic behavior of hydrogels could be easily varied by simply choosing the proper KOH concentration or HEC–DVS ratio. The gels showed ionic conductivities as high as 6 mS cm^−1^.

As a proof of concept, the potential of these CBHs as electrolytes for electrochemical devices like electrochromic (EC) devices and batteries was assessed. On one hand, benefiting from the reaction conditions, ECDs with an all-in-one configuration employing flexible substrates were assembled, providing high transmittance changes (i.e., Δ%T = 55.8%), suitable switching times (ca. 11 s) and appropriate color efficiencies (about 218 cm^2^ C^−1^), as well as proving the flexibility of the device thanks to the mechanical properties of the hydrogel. On the other hand, CBHs were used as electrolytes for printed batteries based on Zn/MnO_2_ chemistry. Taking the advantage of the swelling ability of this hydrogel, the electrolyte was tuned to the battery needs. The printed battery showed a usual discharge profile for this type of battery chemistry and delivered a capacity as high as 1100 µAh (11 mAh g^−1^ Zn; 0.08 mAh cm^−2^; 7.7 Ah cm^−3^) at 50 µA. These results allow demonstrating the feasibility and versatility of cellulose-based hydrogels as suitable, less toxic base electrolytes for electrochemical devices to be implemented in, e.g., smart labels, wearable electronics and wireless sensors. Thus, this study aims to promote further research on the use of well-known hydrogel systems based on biomaterials for more sustainable and cost-effective advanced applications.

## Figures and Tables

**Table 1:** Influence of the cellulose derivatives’ weight ratios on the gel formation and appearance.

Cellulose-Based Hydrogels (CBH) ID	Carboxymethyl Cellulose (CMC)	Hydroxyethyl Cellulose (HEC)	Divinyl Sulfone (DVS)	KOH	Observation
	Wt. %	Wt. %	Wt. %	mM	
**CBH-1**	0	4	2	20	Gelled, release of water
**CBH-2**	1	3			Full gel formed, no release of water
**CBH-3**	2	2			
**CBH-4**	3	1			
**CBH-5**	4	0			Not gelled

**Table 2:** Dependence of gel time, storage modulus (G′), mesh size (ξ) and ionic conductivity (σ) on KOH concentration and HEC to DVS weight ratio.

Gel ID	CMC	HEC	DVS	KOH	Gel Time	G′	ξ	σ (mS/cm)	
	wt. %	wt. %	wt. %	mM	min	Pa	nm	Gel	KOH sol.
CBH-6	2	2	2	10	45	1100	15.52	5.2	0.82
CBH-3				20	12	3100	10.99	4.7	1.63
CBH-7				30	8.3	7300	8.26	5.8	2.41
CBH-8				40	7.4	9100	7.67	6.0	3.25
CBH-9	2	1	3	20	21	1100	15.52	5.0	1.63
CBH-10		3	1		13	3500	10.55	3.3	

## Supplementary Materials

The following are available online at https://www.mdpi.com/2073-4360/12/11/2686/s1, Figure S1: Rheological characterization of CBH-3. Figure S2: Rheological characterization of CBH versus KOH concentration. Figure S3: Rheological characterization of CBH versus the HEC–DVS ratio. Figure S4: Assessment of the feasibility of the CBH for flexible ECDs. Figure S5: Schematic representation of a printed Zinc/MnO2 battery. Figure S6: Galvanostatic discharge curves of printed battery with CBH-3 electrolyte. Figure S7: Electrochemical spectroscopy impedance (Nyquist plots) of CHB-3 and 1M ZnCl2 swelled CBH-3 electrolytes. Figure S8: Digital photographs of the effect of the addition of ZnCl2 (1 M) to 2 wt. % of CMC (left) and to 20 mM of KOH (right). Figure S9: Digital photographs of CBH-3 membranes. Figure S10: Galvanostatic discharge curve at 50 µA of printed battery with a Whatman separator soaked in 1M ZnCl2 electrolyte. Table S1: Transmittance (%) at 550 nm and transmittance changes (∆%T) of ECDs comprising optimized self-standing cellulose-based EC hydrogel at different applied potentials. Table S2: Color coordinates of ECDs comprising optimized self-standing cellulose-based EC hydrogel at bleached (off) and colored states (−2.4 V). Table S3: Bending test of flexible ECDs: transmittance (%) at 550 nm and transmittance changes (∆%T) of flexible ECDs comprising optimized cellulose-based hydrogel before and after bending test (50 cyles).
