# Supplementary material for: Room-Temperature Self-Standing Cellulose-Based Hydrogel Electrolytes for Electrochemical Devices
Source: Polymers (Basel). 2020 Nov 13;12(11):2686. doi: 10.3390/polym12112686 (PMC7696359; doi:10.3390/polym12112686)
Supplement: Supplementary file 1 [file polymers-12-02686-s001.pdf]

Supplementary Materials

Room temperature self-standing cellulose based hydrogel electrolytes for electrochemical devices: Supplementary Electronic Information

Iñaki Gomez *, Yolanda Alesanco, Jose Alberto Blázquez, Ana Viñuales and Luis C. Colmenares *

CIDETEC, Basque Research and Technology Alliance (BRTA), Paseo Miramón 196, 20014 Donostia-San Sebastián, Spain; yalesanco@cidetec.es (Y.A.); ablazquez@cidetec.es (J.A.B.); avinuales@cidetec.es (A.V.)

* Correspondence: igomez@cidetec.es (I.G.); lcolmenares@cidetec.es (L.C.C.); Tel.: +34-943309022

List of figures:

Figure S1. Rheological characterization of CBH-3: (a) time sweep, (b) strain sweep and (c) frequency sweep (same characterization done for all the investigated hydrogels).

Figure S2. Rheological characterization of CBH versus KOH concentration: (a) strain sweeps (linear viscoelastic regime) and (b) frequency sweeps (linear modulus plateau).

Figure S3. Rheological characterization of CBH versus the HEC-DVS ratio: (a) strain sweeps (linear viscoelastic regime) and (b) frequency sweeps (linear modulus plateau).

Figure S4. Assessment of the feasibility of the CBH for flexible ECDs: (a) Digital images of the colored devices comprising optimized cellulose-based EC hydrogel in bended state; (b) Digital images of the ECD on the bleached state (b.1: OFF) and its colored state before (b.2) and after (b.3) 50 cycles of bending; (c) Transmittance spectra of the ECD in its bleached state (OFF) and at its colored state before and after bending.

Figure S5. Schematic representation of a printed Zinc/MnO_2 battery.

Figure S6. Galvanostatic discharge curves of printed battery with CBH-3 electrolyte.

Figure S7. Electrochemical Spectroscopy Impedance (Nyquist plots) of CHB-3 and 1M $ZnCl_2$ swelled CBH-3 electrolytes.

Figure S8. Digital photographs of the effect of the addition of $ZnCl_2$ (1 M) to 2 wt. % of CMC (left) and to 20 mM of KOH (right).

Figure S9. Digital photographs of CBH-3 membranes (a) as made (b) after drying at 70 °C and (c) after swelled with 1M $ZnCl_2$ over 180 minutes.

Figure S10. Galvanostatic discharge curve at 50 μA of printed battery with a Whatman separator soaked in 1M $ZnCl_2$ electrolyte.

List of tables:

Table S1. Transmittance (%) at 550 nm and transmittance changes (Δ%T) of ECDs comprising optimized self-standing cellulose-based EC hydrogel at different applied potentials.

Table S2. Color coordinates of ECDs comprising optimized self-standing cellulose-based EC hydrogel at bleached (off) and colored states (-2.4 V).

Table S3. Bending test of flexible ECDs: transmittance (%) at 550 nm and transmittance changes (Δ%T) of flexible ECDs comprising optimized cellulose-based hydrogel before and after bending test (50 cyles).

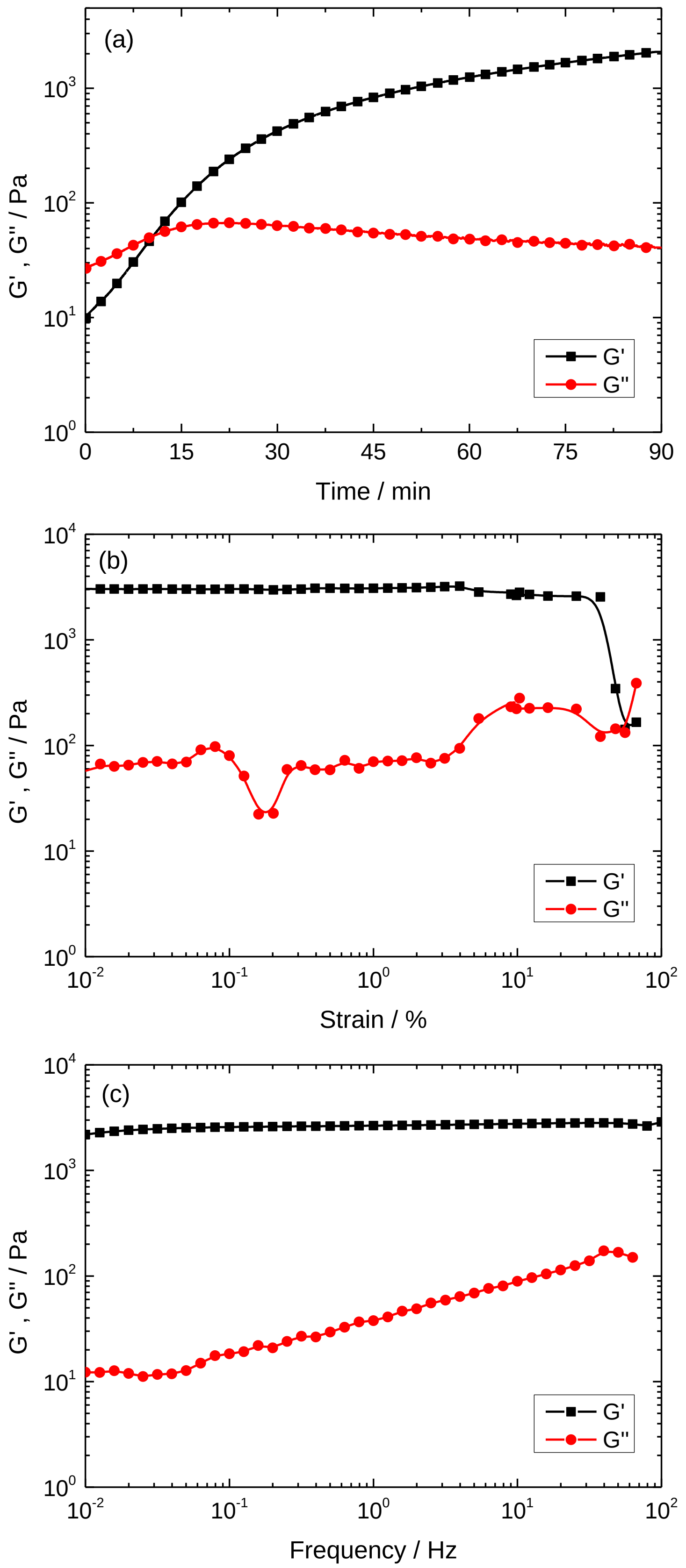

Figure S1. Rheological characterization of CBH-3: (a) time sweep, (b) strain sweep and (c) frequency sweep (same characterization done for all the investigated hydrogels)

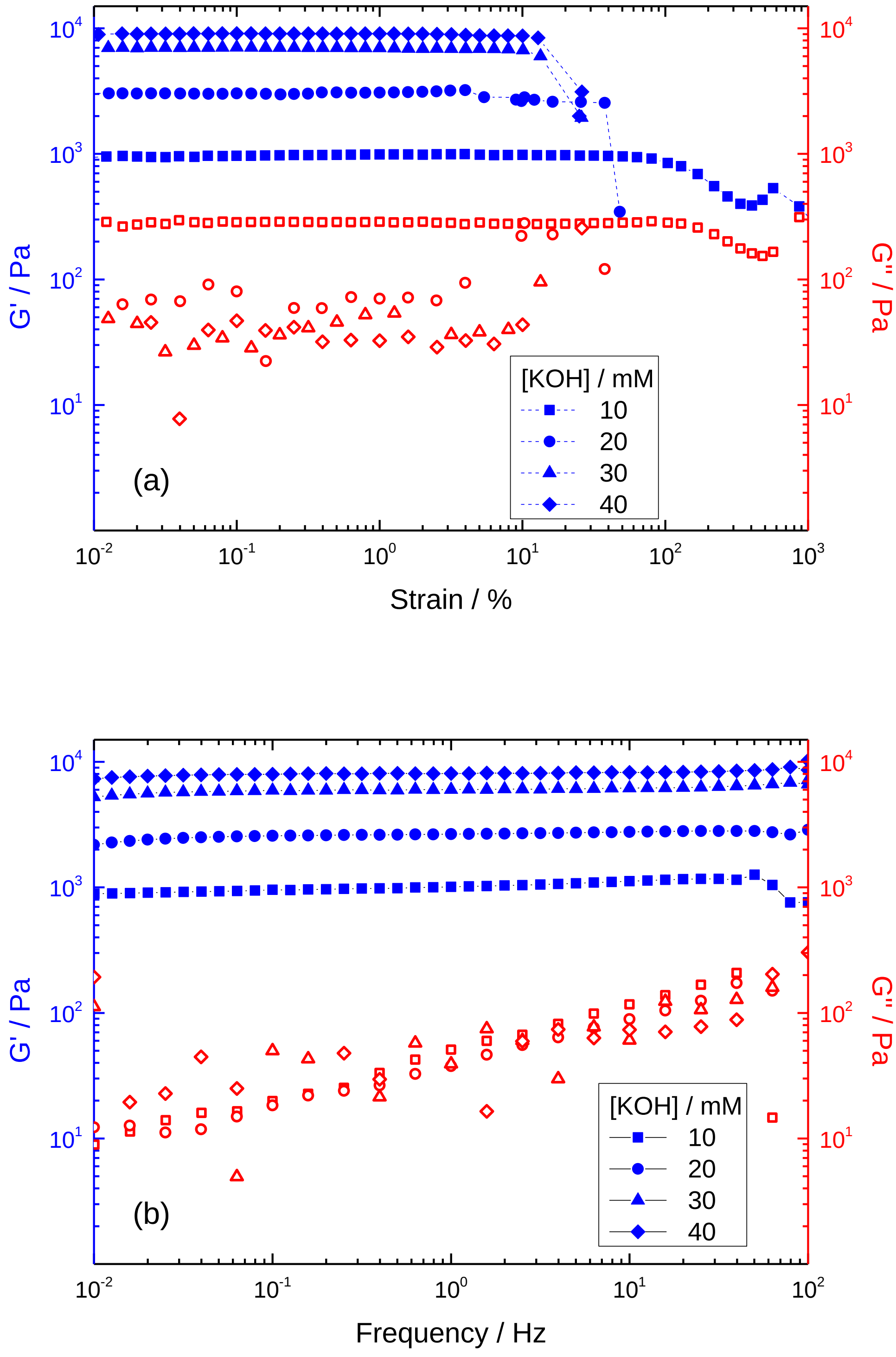

Figure S2. Rheological characterization of CBH versus KOH concentration at a ratio HEC to DVS of 50 : 50: (a) strain sweeps (linear viscoelastic regime) and (b) frequency sweeps (linear modulus plateau).

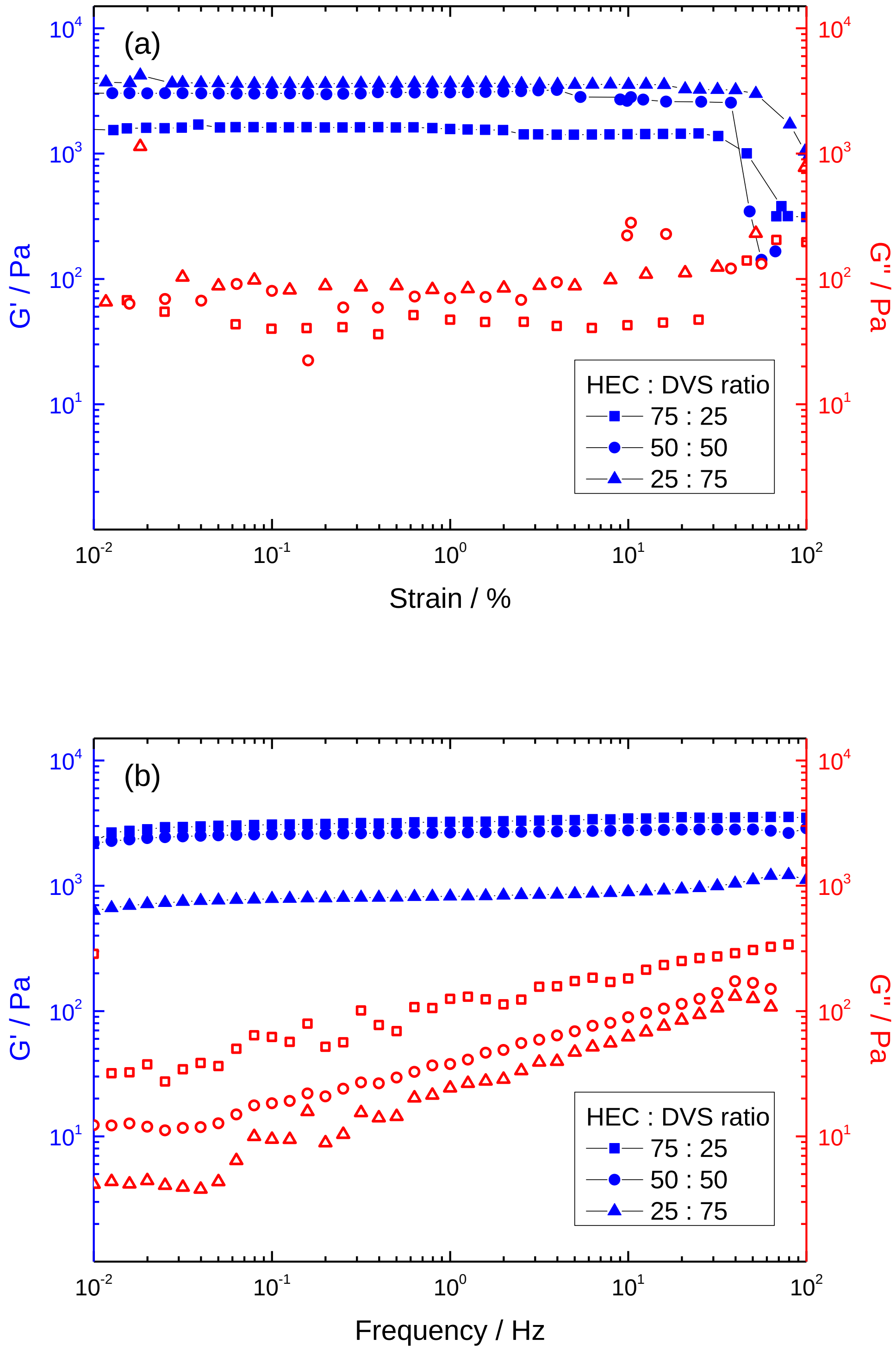

Figure S3. Rheological characterization of CBH versus the HEC-DVS ratio in 20 mM KOH: (a) strain sweeps (linear viscoelastic regime) and (b) frequency sweeps (linear modulus plateau).

Table S1. Transmittance (%) at 550 nm and transmittance changes (Δ%T) of ECDs comprising optimized self-standing cellulose-based EC hydrogel at different applied potentials.

Potential (V)	%T (λ = 550 nm)	Δ%T (λ = 550 nm)
OFF	67.5	-
-1.2 V	38.0	29.4
-1.4 V	30.8	36.6
-2.0 V	25.9	41.6
-2.2 V	16.6	50.8
-2.4 V	11.7	55.8

Table S2. Color coordinates of ECDs comprising optimized self-standing cellulose-based EC hydrogel at bleached (off) and colored states (-2.4 V).

Potential (V)	x [a]	y [a]	Y [a]	L* [b]	a* [b]	b* [b]	Color [c]
OFF	0.316	0.332	68.318	86	0	2	
-2.4 V	0.287	0.215	15.396	46	32	-31	

Table S3. Bending test of flexible ECDs: transmittance (%) at 550 nm and transmittance changes (Δ%T) of flexible ECDs comprising optimized cellulose-based hydrogel before and after bending test (50 cyles).

**Potential (V)**	**%T (λ = 550 nm)**	**Δ%T (λ = 550 nm)**
OFF	64,4	-
ON	26,0	38,4
ON-after bending	25,5	39,0

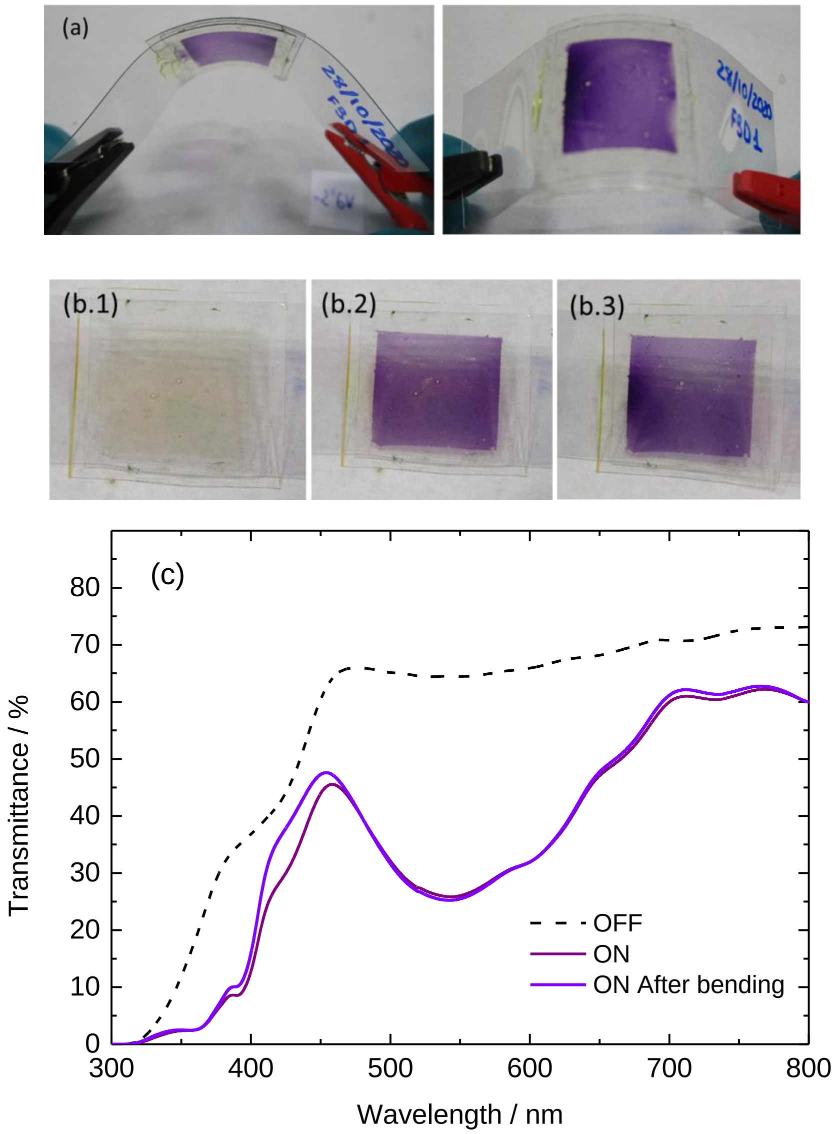

Figure S4. Assessment of the feasibility of the CBH for flexible ECDs: (a) Digital images of the colored devices comprising optimized cellulose-based EC hydrogel in bended state; (b) Digital images of the ECD on the bleached state (b.1: OFF) and its colored state before (b.2) and after (b.3) 50 cycles of bending; (c) Transmittance spectra of the ECD in its bleached state (OFF) and at its colored state before and after bending.

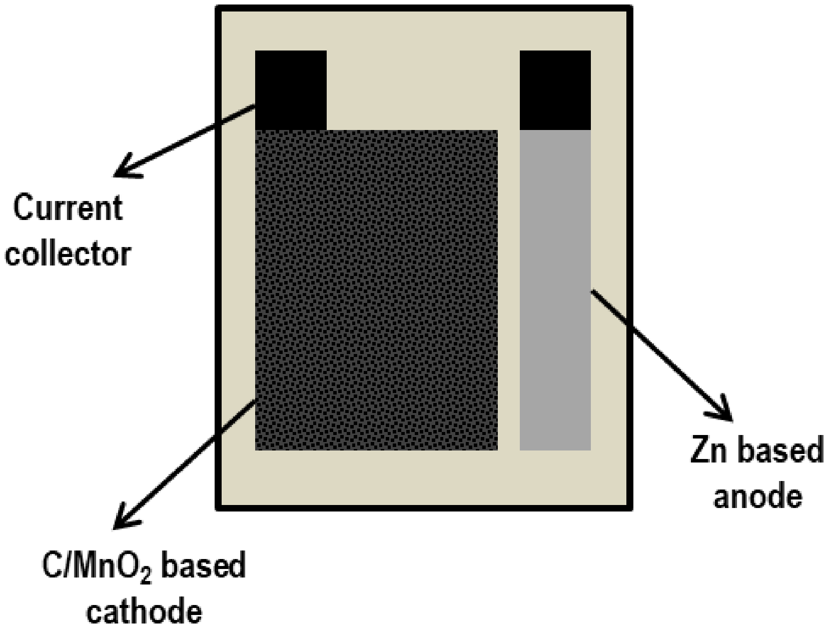

Figure S5. Schematic representation of a printed Zinc/MnO_2 battery.

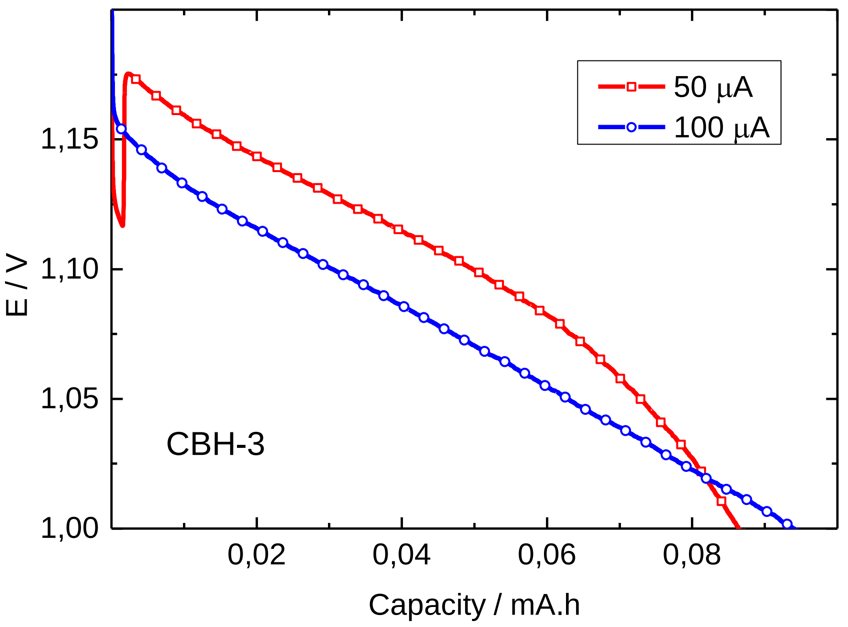

Figure S6. Galvanostatic discharge curves of printed battery with CBH-3 electrolyte.

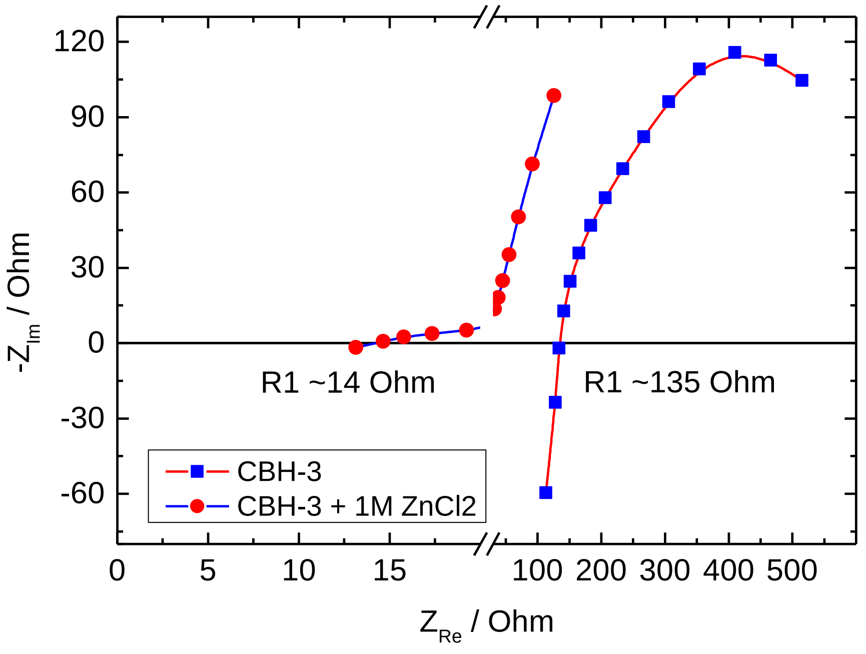

Figure S7. Electrochemical Spectroscopy Impedance (Nyquist plots) of CHB-3 and 1M $ZnCl_2$ swelled CBH-3 electrolytes.

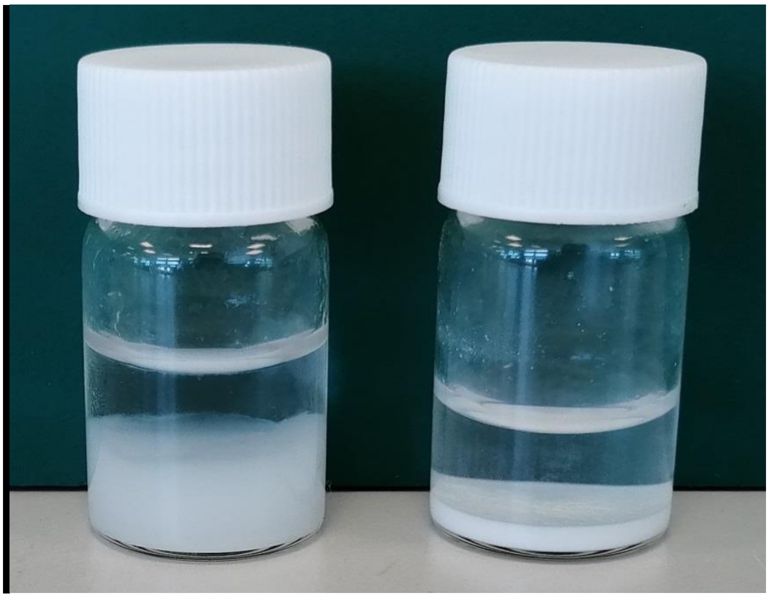

Figure S8. Digital photographs of the effect of the addition of $ZnCl_2$ (1 M) to 2 wt. % of CMC (left) and to 20 mM of KOH (right).

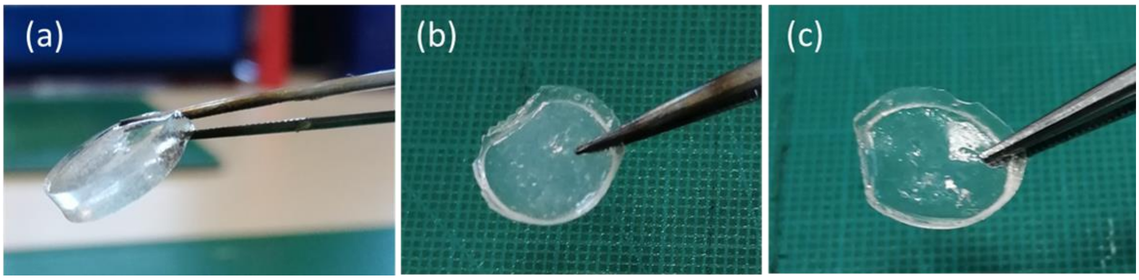

Figure S9. Digital photographs of CBH-3 membranes (a) as made (b) after drying at 70 °C and (c) after swelled with 1M $ZnCl_2$ over 180 minutes.

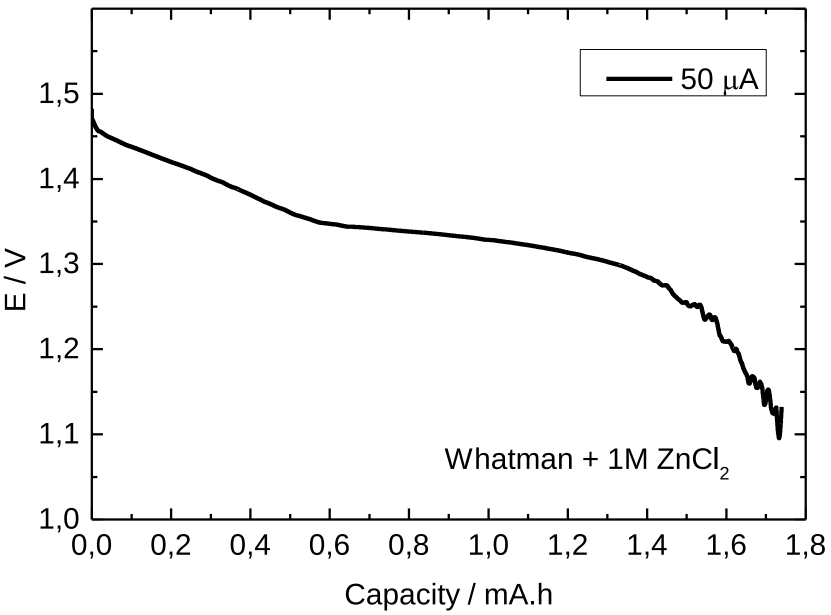

Figure S10. Galvanostatic discharge curve at 50 μA of a printed battery with a Whatman separator soaked in 1M $ZnCl_2$ electrolyte.
